# Effect of ambient lead on progesterone and pregnancy-associated glycoprotein 1 and their relationship with abortion in Zaraibi goats: a field study

**DOI:** 10.1007/s11250-023-03877-w

**Published:** 2024-01-12

**Authors:** Abrar F. Mosaad, Sayed M. El-Nakhla, Ferial H. Abd El-Rasoul, Ahmed M. Shehabeldin, Atef A. Ali, Gamal M. Morsy

**Affiliations:** 1 Sheep & Goat Research Department, Animal Production Research Institute, Agricultural Research Center (ARC), Giza, Egypt; 2 Zoology Department, Faculty of Science, Cairo University, Giza, 12613 Egypt

**Keywords:** Lead, Progesterone, Pregnancy-associated glycoproteins, Abortion, Bioavailability, Zaraibi goats

## Abstract

This study aimed to investigate the impact of ambient lead (Pb) exposure on progesterone (P_4_) and pregnancy-associated glycoprotein 1 (PAG1) and their relationship with abortion in Egyptian Zaraibi goats (*C. hircus*). To achieve this, 40 female goats (does) were mated with highly fertile male goats, resulting in a total of 28 pregnant goats. Eight of them aborted, and each of the 12 pregnant goats gave birth to one kid, whereas the remaining eight gave birth to twins. The levels of PAG1, P4, and Pb in serum were estimated by enzyme-linked immunosorbent assay (ELISA), radioimmunoassay (RIA), and inductively coupled plasma mass spectrometry (ICP-MS) respectively. Statistically, the repeated measure two-way ANOVA, regression analysis, correlation coefficient, and receiver operating characteristic (ROC) curves were applied. The current data demonstrated that the levels of blood Pb in aborted goats were significantly higher than those in non-aborted goats at the early, mid, and late gestations, and this was followed by significant decreases in serum PAG1 and P_4_. Furthermore, there were substantial inverse associations between blood Pb concentration and levels of PAG1 and P_4_, with markedly negative correlation coefficients of − 0.88 and − 0.77, respectively, in aborted goats. The threshold level of Pb required to cause abortion was ≥ 32.08 μg/dl, but for PAG1 and P_4_ were respectively ≤ 0.95 ng/ml and ≤ 0.48 ng/ml. Additionally, threshold levels of ≥ 12.34 ng/ml and ≥ 31.52 ng/ml for P_4_ and PAG1, respectively, were needed to deliver twins. In conclusion, pollution-induced increases in Pb bioavailability resulted in dramatic decreases in P_4_ and PAG1 levels, leading to abortions. PAG1 and P_4_ levels are also key factors in determining whether Zaraibi goats will give birth to twins.

## Introduction

Zaraibi goats are considered one of the most important economic sources of livestock to Egyptian peoples till now (Nowier et al. 2020). Egypt has over 4.3 million goats, the majority of which are Baladi and Barki goats raised for meat production (Hassen and Tesfaye 2014) and Zaraibi goats reared for milk production (Galal 2005). The Egyptian Nubian goat (E. Nubian), known as the Zaraibi or Nubi goat in Upper Egypt of the Arab Republic of Egypt, Zaraibi goats are one of the main ancestors of the common Anglo-Nubian goat (Aboul-Naga et al. 2012).

One of the key components of animal production in Egypt and a significant source of red meat is the goat industry. The amount of goat meat produced in Egypt during the period (2015–2019) was about 30 thousand tons, which represents about 4.14% of the average total production of red meat in Egypt (Hosny et al. 2022).

To increase goat production and population, successful reproduction is essential; hence, it is important to have a thorough understanding of animal physiology throughout the various stages of reproduction (Salve et al. 2016). Reproductive health issues hinder goat breeding development plans (Haile 2014). Reproductive disorders negatively impact goat producers, reducing food production and affecting threatened animal species’ persistence. Major abortions and pregnancy losses due to embryonic mortality constrain gestation in all livestock animals (Yadav et al. 2021). Abortion is a multifactorial phenomenon controlled by many factors, including infectious agents (bacterial, viral, fungal, and protozoan agents, etc. and non-infectious factors such as toxicities, malnutrition, stress, maternal endocrine imbalance, and ambient temperature (Hajiabadi et al. 2022).

Heavy metals in animal feed and water can harm animal health due to their bioaccumulation (Agbugui and Abe 2022; Ghazzal et al. 2022). Exposure to sub-lethal quantities of Pb can negatively affect various biochemical and physiological systems (Elarabany and El-Batrawy 2019). Ruminants are often exposed to toxic environmental toxins, posing a threat to animal health (Gensa 2019; Mridula et al. 2022). These toxins affect various organs, like the reproductive, nervous, respiratory, liver, gastrointestinal, and endocrine systems (Volkov and Ezhkova 2020; Bíreš et al. 1995). They caused poor body conditions, slowed reproduction rates, and cancer due to their mutagenicity, teratogenicity, and carcinogenicity (Bíreš et al. 1995; Dasharathy et al. 2022).

Lead is a reproductive toxin that affects female animals’ reproduction, causing endometritis in ewes (Stoev et al. 1997), decreased fertility in cows )McEvoy and McCoy 1993), poor conception rates, decreased heat detection, and longer service intervals in buffalo cows (El-Tohamy et al. 1997).

Pregnancy detection is crucial for animal production systems to avoid abortion in herds due to unknown causes (Smith et al. 2015). Therefore, the reproductive process for any animal production system must include a crucial stage known as pregnancy detection for decisions on rebreeding or culling non-pregnant females. Early, accurate, and practical methods are needed for reproductive performance improvement (Arashiro et al. 2018). Various methods, including abdominal palpation, radiography, ultrasonography, and hormone detection, are being used in small ruminants with variable diagnostic accuracy. This helps in making informed decisions on rebreeding or culling non-pregnant females.

Numerous methods for small ruminant pregnancy detection have been developed to optimize reproductive performance in goats (Karadaev 2015). The ideal pregnancy test should have high sensitivity, specificity, and simplicity in conducting under field conditions (Pohler et al. 2016).

Pregnancy-associated glycoproteins (PAG), a large family of inactive aspartic proteinases, are only secreted by mono- and bi-nucleate trophectoderm cells (Xie et al. 1991). Since PAG only has ruminant placental origin, it is thought to be a better appropriate biomarker for pregnancy in goats (Roberts et al. 2017). Bovine species have had 22 PAG genes (boPAG-1 to boPAG-22) cloned and fully sequenced (Garbayo et al. 2008). Not all PAGs are detectable at the same period of gestation; some arrive earlier and others later (Green et al. 2000). While some (bPAG-1, bPAG-6, and bPAG-7) are present from the middle to the end of pregnancy, others (bPAG-4, bPAG-5, and bPAG-9) start to appear about day 25 but are missing in the last stages of pregnancy (Green et al. 2000).

Zamfirescu et al. (2011) reported that progesterone (P_4_) and pregnancy-associated glycoproteins (PAGs) were considered as laboratory tools for pregnancy detection and observed that the quantitative measurement of (PAGs) can be used to confirm early gestation in goats.

The current study is part of an integrated research project to identify the environmental factors that may be the direct and/or indirect cause of the frequent and noticeable abortion of female goats on many animal farms in Egypt. This resulted in a sharp and noticeable decline in livestock and in the number of goats, resulting in a reduction in the meat of female Zaraibi goats in animal farms. To achieve this goal, physiological parameters of progesterone (P_4_), pregnancy-associated glycoprotein 1 (PAG1), and ambient ionic pollutants of lead (Pb) in the blood were measured in pregnant goats. The regression and correlation analyses were performed to determine the significant relationships and correlation coefficients of ambient Pb ions with gestation stages and serum PAG1 and P_4_ levels to clarify their effect on abortion.

## Materials and methods

### Management of animals

The animals were kept as part of the flock of the Animal Production Research Institute (APRI) and Agriculture Research Center (ARC) Sakha Experimental Station in Kafr El-Sheikh governorate (31.089°N, 30.951°E). The current study comprised 40 healthy and disease-free multiparous estrous-cycle native Egyptian Zaraibi goats (*C. hircus*). Throughout the trial, the animals were group-housed and maintained in a semi-intensive management system with uniform dietary conditions (65% undercoated cottonseed cake, 11% rice straw, 18% wheat bran, 3% molasses, 2% limestone, and 1% salt), 660 g/head/day and free access to water and salt blocks. This portion of the field study lasted for five months, from May to September 2018.

A single vasectomized male Zaraibi goat (an infertile male) was introduced twice daily at 8 a.m. and 4 p.m. to detect the estrus phase of the does. Five viable mature fertile male Zaraibi goats (bucks) were used for mating estrus females. Mating was allowed to occur spontaneously for 45 days, and pregnant goats were identified using ultrasound and ultrasonography.

Ultrasound investigations were conducted using ultrasound (Renco Preg-Tone), according to Quintela et al. (2012). Transrectal ultrasonography was performed on all animals until the 60th day of pregnancy (ESOATE Pie Medical Aquila Pro-Vet + probe, 6.0 MHz LA Rectal Veterinary Transducer), as described by Padilla-Rivas et al. (2005). Ultrasonography of the pregnant uterus revealed an anechoic embryonic vesicle (black) encircling the echoic (white) elongated streak (foetus), which extended across more than half of the fetal fluid. Ultrasonography revealed that 28 of the 40 goats were pregnant, while the remaining 12 did not become pregnant and were therefore excluded from the study. Eight goats aborted during pregnancy, while the remaining 20 goats gave birth to 12 singles and eight twins. As a result, there were 12 single and eight twin pregnancies.

Prior to the trials, all goats had received vaccinations against the most contagious diseases. The Institutional Animal Care and Use Committee (IACUC) of Cairo University authorized the care and handling of animals under the permit CU/I/S/96/17. All measurements performed in compliance with the veterinary standards were approved by the Animal Ethics Committee of the Institute.

### Blood sampling

Blood samples were obtained by jugular venipuncture. The blood was collected in non-heparinized tubes at room temperature and then centrifuged at 3000 × g for 15 min at 4 °C to separate the serum that was stored at − 20°C until measurements of lead (Pb), pregnancy-associated glycoprotein 1 (PAGs), and progesterone (P_4_) levels in the serum of aborted and non-aborted goats.

### Hormone assay

#### Pregnancy-associated glycoprotein 1

The estimation of pregnancy-associated glycoproteins (PAGs) levels was quantified by enzyme-linked immunosorbent assay (ELISA) using a bovine pregnancy-associated glycoproteins 1 (PAG1) ELIZA kit using kits from Shanghai Coon Koon Biotec Co., Ltd., Room 1408, 1687 Chang Yang RD, Shanghai, China. Cat No. CK-bio-18624, Standard Curve Range: 1–48 ng/ml; sensitivity: 0.1 ng/ml). The assay was conducted according to the procedures described in the enclosed catalog, and an automatic photometer plate reader was used for readings of absorbencies. The intra-assay precision (CV%) was less than 10%. The inter-assay precision (CV%) was < 15%. The computed level of PGA1 in serum was expressed as nanograms per milliliter (ng/ml).

#### Progesterone assay

Serum progesterone (P_4_) levels were measured by radioimmunoassay (RIA) using a DIA source PROG-RIA-CT Kit (KIP1458; DIA source ImmunoAssays S.A., Rue du Bosquet, 2, B-1348 Louvain-la-Neuve, Belgium).the principle of this method was described by (Alper et al. 1987). The computed concentration of progesterone (P4) in serum was expressed as nanograms per milliliter (ng/ml).

### Lead assay

The lead (Pb) ion serum content was estimated using inductively coupled plasma mass spectrometry (ICP-MS), according to(Morsy et al. 2016). The sera were completely digested by concentrated nitric acid that evaporated after complete digestion until yellowish-white ash appeared on the wall of the test tube. The precipitate was dissolved in 3 ml HCl and diluted with deionized water. The estimated lead (Pb) level in the serum was expressed as μg/dl.

### Statistical analysis

The Kolmogorov-Smirnov test confirmed that the current data were normally distributed; hence, a parametric statistical analysis was used. Accordingly, the repeated-measures greenhouse two-way analysis of variance (ANOVA) due to the non-homogeneity of the raw data was applied to clarify the significant changes in the studied dependent variables of Pb, PAGs, and P4 contents as direct responses of the independent variables of the pregnancy intervals (28, 46, 60, 88, 108, 128, and 148 days) and, in turn, the effect of these parameters on cases of birth (abortion and delivery of one or two fetuses). In addition, post hoc analysis of variance (ANOVA) of Schiff’s test was used to compare dependent values.

Regression analysis and correlation coefficients were computed to fit the relationship between the interval pregnancy period (independent variable) and the dependent variables, as well as the association between the levels of Pb ions in the serum and the concentrations of P_4_ and PAG1 in the serum of aborted goats. The levels of studied parameters at, above, or below which abortion occurred were identified using diagnostic statistical analysis of receiver operating characteristic (ROC) curves. IBM Statistical Package for the Social Sciences, version 28, was used to analyze the data.

## Results

**Photo 1:**
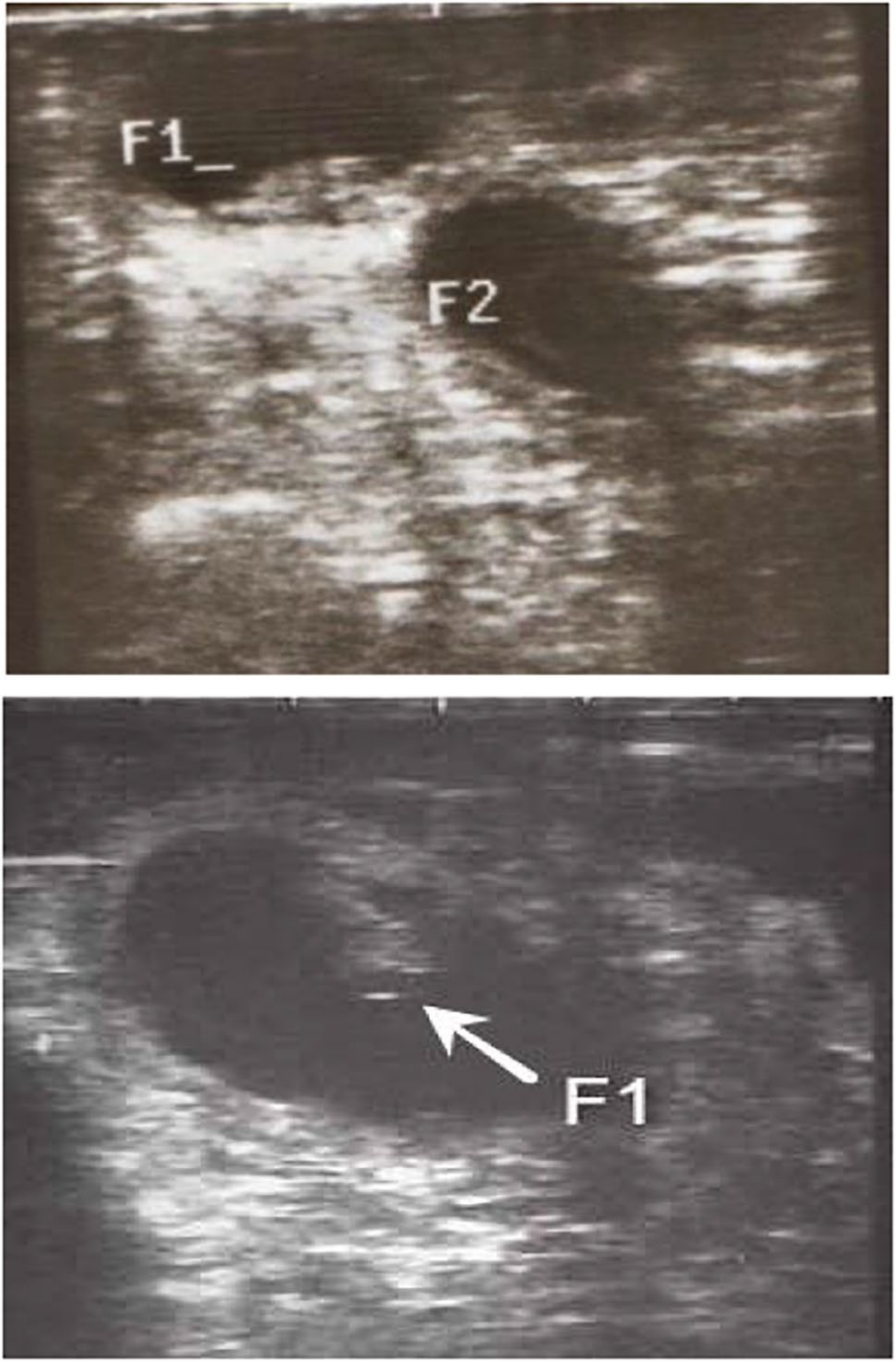
Transrectal ultrasonographic photos of two pregnant does. One had a single embryo at day 32 while the other had twins embryos at day 26

The ultrasonographic images of Zaraibi goats showed that 28 (70%) of the 40 (100%) females were pregnant, while 12 females (30%) were not. On days 30, 39, 52, 63, 72, 76, 90, and 101, respectively, eight of the 28 pregnant goats miscarried and lost their fetuses (28.6%), whereas the other 20 goats (71.4%) carried their pregnancies to term. Of the 20 pregnant goats that were photographed, eight (40%) had uteruses that held twins, while the uteruses of the remaining 12 females (60%) each carried a single kid (Photo 1).

Repeated-measures two-way analysis of variance demonstrated that the pregnancy stage had a significant effect on the levels of serum Pb, PAG 1, and P_4_ in pregnant Zaraibi goats, which in turn had a substantial effect on abortion and the number of kids born (Table 1).

**Table 1:** The repeated measures greenhouse two-way ANOVA table that analyzes the changes of the levels of serum lead (Pb, μg/dl); the pregnancy-associated glycoprotein1 (PAG1, ng/ml), and progesterone (P_4_, ng/ml) contents in pregnant Zaraibi goats as a response to the pregnancy stages and their effect on the birth cases (abortion or non-abortion)

Parameters	Source	SS	df	MS	*F*_calculated_	*P*-values
Pb	PP	285.851	3	89.325	22.241	*P* < 0.0001
	PP*BC	633.278	6	98.946	24.637	*P* < 0.0001
	Error	321.309	80	4.016		
PAG1	PP	3518.367	3	1112.609	114.180	*P* < 0.0001
	PP*BC	4001.279	6	632.659	64.926	*P* < 0.0001
	Error	770.355	79	9.744		
P_4_	PP	38.328	3	12.325	14.729	*P* < 0.0001
	PP*BC	186.284	6	29.951	35.794	*P* < 0.0001
	Error	65.053	78	0.837		

*SS* sum of squares, *df* degree of freedom, *MS* mean of squares

*F*_calculated_: the computed *F*-value of the data

PP*BC: interaction of pregnancy period intervals with the birth cases (abortion, single, or twins)

*P* < 0.0001: significant effect at *α* = 0.0001

According to the post hoc analysis of variance (ANOVA) for Scheffe’s test, the serum Pb content of aborted goats was significantly higher than that of goats that delivered one or two kids throughout all pregnancy periods, whereas in goats that delivered a single kid or twins did not differ (Table 2). The gestation stages (28, 46, 60, 88, 108, 128, and 148 days) had a significant direct exponential relationship with the levels of Pb in aborted goats and were accompanied by marked positive correlation coefficients of + 0.98 (Table 2).

**Table 2:** Changes in serum lead (Pb, μg/dl), pregnancy-associated glycoproteins (PAG1, ng/ml), and progesterone (P_4_, ng/ml) contents of Zaraibi goats who aborted and those who gave birth to a single kid or twins during pregnancy stages (28, 46, 60, 88, 108, 128, and 148 days)

Pregnancy stages	Lead (Pb, μg/dl)			PAG1 (ng/ml)			Progesterone (P_4_, ng/ml)		
	Aborted	Single	Twins	Aborted	Single	Twins	Aborted	Single	Twins
28	33.042 ± 1.242	9.74 ± 0.992*	9.02 ± 01.358*	2.963 ± 0.350	9.492 ± 0.286*	27.231 ± 0.638*^■^	3.417 ± 0.322	8.832 ± 0.263*	11.024 ± 0.322*^■^
46	34.681 ± 0.967^**a**^	9.46 ± 0.991*	9.74 ± 0.357*	1.729 ± 0.580	12.976 ± 1.974*^a^	39.760 ± 1.921*^■**a**^	2.598 ± 0.310^**a**^	10.291 ± 0.253^a^*	11.258 ± 0.310*^■^
60	35.962 ± 1.406^**ab**^	9.14 ± 1.288*	9.90 ± 0.597*	1.216 ± 0.601	19.085 ± 1.307*^ab^	39.939 ± 1.184*^ab■^	1.759 ± 0.280^**ab**^	10.729 ± 0.228^a^*	11.639 ± 0.280*^■^
88	37.072 ± 1.060^**abc**^	10.16 ± 0.957*	8.98 ± 0.859*^■^	0.986 ± 0.197^**a**^	34.013 ± 2.650*^abc^	44.567 ± 1.813*^abc■^	0.837 ± 0.214^**abc**^	10.833 ± 0.338^a^*	14.735 ± 0.414*^abc■^
108	38.665 ± 1.304^**abcdc**^	9.713 ± 1.182*	9.95 ± 0.223*	1.005 ± 0.433^**a**^	29.782 ± 1.353*^abcd^	46.354 ± 2.582*^abc■^	0.385 ± 0.186^**abcd**^	11.183 ± 0.234^ab^*	14.566 ± 0.286*^abc^
128	40.332 ± 2.344^**abcde**^	9.95 ± 0.869*	9.13 ± 0.661*	0.999 ± 0.247^**a**^	28.983 ± 1.610*^abcd^	41.617 ± 1.963*^abcd■^	0.372 ± 0.110^**abc**^	10.402 ± 0.172^ae^*	12.454 ± 0.210*^abcde■^
148	44.781 ± 2.606^**abcdef**^	8.96 ± 1.704*	9.18 ± 0.862*	0.961 ± 0.259^**a**^	28.632 ± 2.538*^abcd^	32.008 ± 1.629*^abcdef■^	0.345 ± 0.197^**abc**^	9.572 ± 0.160*^abcdef^	12.734 ± 0.197*^abcde■^
*R*	*y* = 25.51*e*^0.003*x*^			*y* = 21.961*x*^−0.656^			*y* = 6.0642*e*^−0.022*x*^		
*r*		+ 0.98*			− 0.78*			− 0.94*	

Data represented as an average ± SEM

The symbols * and ^■^ indicate a significant difference (*P* < 0.05) in comparison with the corresponding aborted goats and those that birth a single kid, respectively

In the same column, letters a, b, c, d, e, and f indicate a significant difference (*P* < 0.05) in comparison with those at pregnancy stages of 28, 46, 60, 88, 108, 128, and 148 days of gestation, respectively

*R* regression equation

r*: indicated the correlation coefficient

The levels of PAG1 and P_4_ in the serum of goats that gave birth to a single kid or twin were substantially greater than those in goats that had an abortion at all corresponding stages of pregnancy (Table 2). Additionally, PAG1 and P_4_ concentrations in twin-bearing goats were significantly higher than those in single-bearing goats at all stages (Table 2). As shown in Table 2, according to the regression analysis and correlation coefficient, in aborted goats, the levels of serum PAG1 and P_4_ content exhibited a significant inverse power and exponential relationship with the gestational stages and were associated with a significant negative correlation coefficient of − 0.78 and − 0.94, respectively (Table 2), that is, the levels of PAG1 and P_4_ decreased with increasing gestational stage time.

**Fig. 1:**
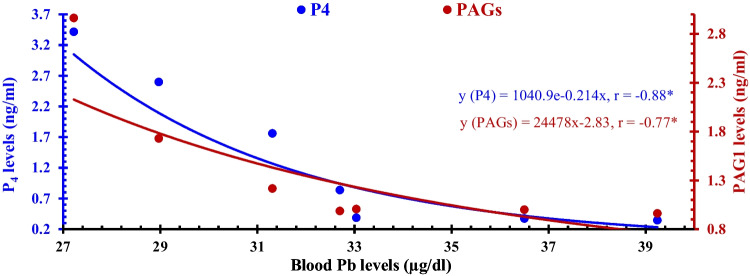
The relationship between levels of lead (Pb, μg/dl) with each of the progesterone (P_4_, ng/ml) and pregnancy-associated glycoprotein (PAG1, ng/ml) content in the sera of aborted Zaraibi goats. The symbol * indicates a significant correlation coefficient between the studied parameters. The letter *x* indicated the levels of Pb ions in sera, whereas the letter *y* is the levels of PAG1 and P_4_ throughout the gestation stages

As shown in Fig. 1, the serum Pb content of aborted goats exhibited a significant inverse power relationship with the concentrations of PAG1, and this was accompanied by a significant negative correlation coefficient of − 0.88, whereas with levels of P_4_ showed a significant inverse exponential association with a significant correlation coefficient of − 0.77.

**Fig. 2:**
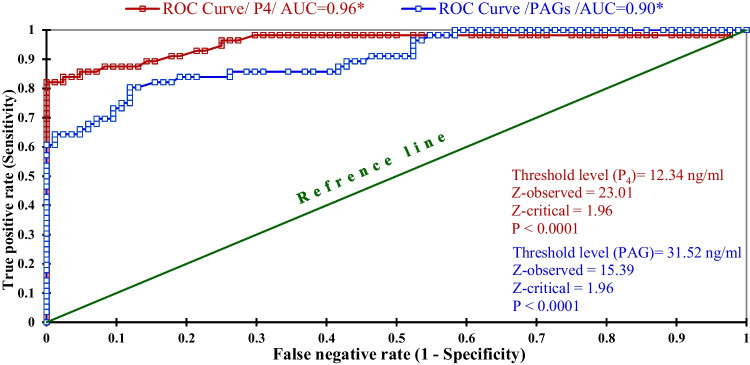
The receiver operating characteristic (ROC) curve determines the threshold level of serum P_4_ (ng/ml) and PAG1 (ng/ml) required for twin pregnancy. The area under the curve (AUC) and its statistics are represented

As seen in Table 3, the receiver operating characteristic (ROC) analysis revealed that the threshold levels of Pb, P_4_, and PAG1 in the serum of aborted goats were ≥ 32.08 μg/dl, ≤ 0.48 ng/ml, and ≤ 0.95 ng/ml, respectively, with a significant area under curves (AUC) of 1.00 (Table 3). This means that the serum Pb level of ≥ 32.08 μg/dl will cause abortion, but below this value will not, and vice versa for P_4_ and PAG1 ≤ 0.48 ng/ml and ≤ 0.95 ng/ml will induce abortion. In addition, as shown in Fig. 2, the threshold values of P_4_ and PAG1 required to induce give birth to twins were ≥ 12.34 ng/ml and ≥ 31.52 ng/ml with significant excellent AUC of 0.96 and 0.90, respectively.

**Table 3:** Threshold values (threshold values), sensitivity (Sen), specificity (Sp), positive (PPV) and negative (NPV) predictive values, accuracy (%), and area under the curve (AUC) of the blood lead (Pb, μg/dl), pregnancy-associated glycoprotein1 (PAG1, ng/ml), and progesterone (P_4_, ng/ml) contents of aborted Zaraibi goats throughout pregnancy stages

Parameters	Threshold values	Sen (%)	Sp (%)	PPV (%)	NPV (%)	Se + Sp (%)	Accuracy (%)	AUC	*P*-value^■^
Pb^1^	32.08	1.0	1.0	1.0	1.0	2.0	1.0	1.0	*P* < 0.001
PAG1^2^	0.95	1.0	0.92	1.0	1.0	2.0	1.0	1.0	*P* < 0.001
P4^2^	0.48	1.0	1.0	0.98	1.0	2.0	1.0	1.0	*P* < 0.001

^1^The test is positive if the levels of desired parameters are ≥ the threshold value

^2^The test is positive if the levels of desired parameters are ≤ threshold value

^■^Significant effect at *α* = 0.001 (*P* < 0.001)

## Discussion

The current findings showed that the bioavailability of blood lead (Pb) levels in aborted goats was significantly higher than that of non-aborted goats at most pregnancy stages. Even though Zaraibi goats were supported by veterinary care under livestock breeding management, laboratory measurements affirmed the presence of Pb in the blood of all non-aborted and aborted goats in varying proportions, indicating that their environment was lead-contaminated. Most toxicologists and environmental pollution experts believe that the presence of Pb in most mammalian tissues, including blood, is normal and not unusual, but in proportions consistent with the permissible limit decided by UNESCO (WHO 1987). This clarifies and explains the Pb ion bioaccumulation in most tissues, including blood, of both aborted and non-aborted goats because of Pb environmental exposure (Azeh Engwa et al. 2019) as in our current findings.

The route of exposure, the physiochemical characteristics, and the toxicokinetic of the Pb molecule, as well as an individual’s age and nutritional status, all have an impact on how much lead is absorbed (Morrow et al. 1980). The levels of free Pb ions reaching systemic blood circulation, because of their absorption via all routes of exposure, are called bioavailability of Pb. According to previous studies, 40% of Pb that is inhaled is deposited in the lungs, and deposited in the lower respiratory tract absorbs almost entirely (Morrow et al. 1980). The duodenum is where Pb is largely absorbed during digestion; however, age and nutritional state can have a significant impact on how quickly lead is absorbed (Graziano et al. 1996). Pb can also be absorbed via healthy skin (Wright et al. 2003). Accordingly, about 95% of the available lead is transported and distributed those ions to all tissues (Stauber et al. 1994) .

In systemic circulation, the bioavailable Pb attaches to hemoglobin in erythrocytes and is then easily transferred to soft tissues such as the kidney, liver, reproductive organs (ovaries, placenta, etc.), and central nervous system (Goutam Mukherjee et al. 2022). Pb accumulates and is stored largely in bones and teeth after redistribution, accounting for up to 90% of the total Pb body burden (Barry 1975). Pb has a half-life of 30 days in blood and most soft tissues, but a half-life of up to 25 years in bone (Hu et al. 1998). Pb produced from bones is a significant endogenous exposure route and/or source that can contribute up to 50% of the blood Pb levels in the absence of exogenous exposure, physiologically, Pb is easily absorbed by the fetus through the placenta and builds up in breast milk (Gulson et al. 1998). On the basis of toxicokinetics, urine and/or biliary clearance are the main pathways by which ingested Pb is expelled from the body via Phase I and II biotransformation; however, in goats and most models of mammalian organisms, biliary clearance outpaces urine excretion of Pb (Rădulescu and Lundgren 2019). Accumulating research also suggests that kids excrete ingested Pb at a slower pace than adults, which may contribute to extended retention durations in kids (Stauber et al. 1994)

Our current data show that the levels of Pb ions in the serum of non-aborted goats were much lower than those of aborted goats. This demonstrates that abortion is entirely reliant on the concentration of Pb ions in goat serum, as well as their bioaccumulation in their tissues, particularly the ovary, placenta, and liver (Canaz et al. 2017). Statistically, the serum Pb concentration threshold value to trigger abortion was ≥ 32.08 μg/dl. This means that the Pb concentration required to induce abortion was ≥ 32.08 μg/dl, implying that Pb concentrations below this level will not cause abortion as demonstrated in our non-aborted goats. This interpretation is reinforced by the fact that blood Pb ion levels in mothers at all stages of pregnancy were substantially below the threshold level and failed to disturb levels of progesterone (P_4_) and pregnancy-associated glycoprotein1 (PAG1) and consequently allowing the pregnancy to continue to term as will discuss below.

In aborted goats, according to the current results, the serum Pb contents were significantly higher than those of non-aborted goats at all the gestation stages. In addition, there was a significant exponential direct relationship between the gestational stages (28, 46, 60, 88, 108, 128, and 148 days), and the levels of Pb ions content, and this was accompanied by a significant positive correlation coefficient. Accordingly, this relationship confirmed the existence of vital continued accumulations of Pb in the serum of aborted goats and consequently in soft tissues of the reproductive system especially the ovaries and placenta (Massányi et al. 2020).

Pregnancy-associated glycoproteins (PAGs), in mammals including goats, are a group of glycoproteins mainly produced by the trophoblast cells of the placenta of mammals. PAGs have been shown to be useful for identifying the presence of vital embryos and for pregnancy follow-up monitoring, particularly in bovine, goats, and other dairy animals (Barbato et al. 2022; Filho et al. 2020). In ruminants, PAGs are synthesized in the mono- and binucleate cells of the trophectoderm and released into maternal blood circulation where they can be quantified (Zoli et al. 1992). PAG1 have been identified and immunolocalized as part of the discoidal-type placenta in some mammalian species (Panasiewicz et al. 2019).

Several studies on goats have linked high PAG concentrations to a decrease in the activity of polymorphonuclear neutrophils (Dosogne et al. 1999), implying that trophoblast PAG production, influencing maternal immunological status, could be a mechanism by which the conceptus protects itself from rejection. PAGs, as stated by Austin et al. (1999), play a hormonal role in the release of granulocyte chemotactic protein-2 (GCP-2), an α-chemokine whose production is stimulated by interferon-τ (IFN-τ) in early pregnancy (Barbato et al. 2022). As a result, IFN-τ and PAGs would play a similar role in the activation of this chemokine, which appears to be implicated in the start of pregnancy. As a result, PAGs have been proposed as a luteotropic component of the placenta (Xie et al. 1994).

Our present results demonstrated that the PAG1 content of goats who gave birth to twins was significantly higher than those who gave birth to a single kid. PAG1 levels in maternal circulation are higher in twin-bearing goats than in single-fetus goats (González et al. 2000; Sousa et al. 1999), and they are also higher (about ten times) in inter-specific pregnancies than in normal intra-specific gestation (Morecroft et al. 2015), which is consistent with our findings. González et al. (2000) found that the goat that delivered twin fetuses had higher PAG concentrations than those that delivered a single fetus. Moreover, in native North Moroccan goats. Chentouf et al. (2008) observed statistical differences between goats carrying one or two fetuses. Vasques et al. (1995) proposed the relationship between fetal growth rate and PAGs increase during pregnancy in cows as the important decline in PAG1 was reflected by stopped trophectoderm development. Additionally, the successive monitoring of PAG1 in goats also enables the identification of trophoblastic activity disorders that result in fetal death (Zarrouk et al. 1999; Batalha et al. 2001; Faye et al. 2004).

As observed in our results, PAG1 are typically detectable in maternal blood starting from around day 28 of pregnancy in cattle (Barbato et al. 2022). The levels of PAG1 increase as the pregnancy progresses and can reach peak levels at different time points depending on the species and individual animal. Different goat breeds may have variations in their PAG profiles, and some breeds may have higher or lower levels of PAGs compared to others (Morecroft et al. 2015).

Progesterone (P_4_) is a steroid hormone that plays a crucial role in the regulation of female reproductive physiology, such as ovulation, implantation, pregnancy maintenance, and lactation (Kolatorova et al. 2022). It exerts its effects by binding to progesterone receptors (PRs), which are expressed in various tissues, such as the uterus, mammary gland, brain, and bone (Dinny Graham and Clarke 1997). During pregnancy, progesterone production is essential for the maintenance of gestation (Arck et al. 2007). The corpora lutea, formed on the ovary after ovulation, produce progesterone in goats (Gaafar et al. 2005). The progesterone levels are highest during mid-pregnancy and gradually decline towards parturition (Convey 1974). It is involved in regulating the estrous cycle and preparing the uterus for pregnancy. It helps to maintain the uterine environment required for successful implantation and embryonic development (Lonergan 2011). Progesterone levels in goats can be used for pregnancy diagnosis and determination. Low progesterone levels can indicate that a doe is not pregnant, while high levels alone do not confirm pregnancy but rather indicate the presence of progesterone (Rawlings and Ward 1977). In addition to its role in reproduction, progesterone also plays a crucial role in synchronizing estrus in goats. Progestogens, which are synthetic derivatives of progesterone, have been used for estrus synchronization in goats (Rawlings and Ward 1977). They help to regulate and control the timing of estrus, allowing for more controlled breeding practices. P_4_ plays an important role in uterine growth promotion and myometrium contractility suppression, oocyte maturation, implantation facilitation, and pregnancy maintenance in the uterus and ovary (Huang et al. 2005). It provided the lobular-alveolar development in the mammary gland to prepare for milk production and reduce milk protein synthesis before parturition (Woo and Shadel 2011).

Our results revealed that the levels of progesterone (P_4_) in the peripheral plasma of pregnant goats increased after mating and remained high from day 28 to day 148 of pregnancy. These findings are consistent with those of Thorburn and Schneider (1972) who found that throughout early pregnancy, plasma progesterone concentrations remain constant from day 8 to day 60, then increase between days 60 and 70, and remain stable until just before parturition. In the pregnant goat, the ovary is the primary location of progesterone production, while production by the placenta is minimal and unlikely to alter the level of this hormone in maternal circulation.

In the present results, levels of PAG1 and P_4_ produced and released by the placenta and corpus luteum, respectively, were sufficient for pregnancy maintenance in Zaraibi goats. According to Sawada et al. (1994), the levels of P_4_ and PAG1 in the serum began to rise after 10 days of mating and persisted up until 140 days of pregnancy before rapidly declining one day before parturition. This is consistent with our current results, with the difference that each of the PAGs and P_4_ reached their highest average on day 88 after mating, then began to decrease slowly and gradually until the date of birth. On the other hand, the PAG and P_4_ levels in our data were different from those reported by González et al. (2004) and Chentouf et al. (2008). We attribute this to the homologous or heterologous goat breed variants that affect P_4_ synthesis, and this reflects the genetic strategy for maintaining a pregnancy under severe conditions (Sousa et al. 1999). PAGs and P_4_ synthesis differ by breed, which reflects a genetic strategy for maintaining a pregnancy under the severe conditions of our Egyptian farm, according to their current levels.

Physiologically, the reactive oxygen species (ROS), which are produced during pregnancy because of metabolic changes in the mother and fetus, are required for the proliferation, differentiation, and maturation of developing cells. This is because the development of the fetal organs during pregnancy requires an appropriate supply of nutrients and oxygen (Bak and Roszkowski 2013).

The placenta of the mother is dyspeptic with mitochondria, which are the primary source of energy since they create and release pro-oxygenates, earning them the moniker “ROS factories” and/or “powerhouses.” The superoxide anion radical, which is formed in vast amounts, is a generator of more reactive oxygen, such as hydrogen peroxide and hydroxyl free radicals. Their production increases as the pregnancy progresses, which is mostly due to an increase in placental mass (Toboła-Wróbel et al. 2020).

During normal pregnancy, the phenomenon of the mother’s immunological tolerance to the fetus’ antigens, which permits the kid to develop in the uterus despite the pregnant female’s ability to reject the foreign antigen, is a crucial factor in a pregnancy that is progressing normally (Toboła-Wróbel et al. 2020). The creation of ROS is reduced in a normally functioning pregnant organism due to the reduced activities of the immune system (Moore et al. 2019). Low levels of ROS work physiologically as a defensive mechanism against pathogenic pathogens (Puertollano et al. 2011) as in non-aborted goats as mentioned above.

High bioaccumulation of serum Pb content reflects its high concentrations in various tissues; they exacerbate oxidative stress in tissues because of the overproduction of ROS by direct action in the mitochondrial electron transport chain, resulting in cellular peroxidation of lipids, proteins, and DNA (Belyaeva et al. 2008), resulting in a cycle of cellular or molecular damage (Bouayed and Bohn 2010) and inflammation in the placenta, which can affect the synthesis and secretion of PAGs and other hormones Mason et al. 2014). On the other hand, Pb accumulates in the tissues of the fetus throughout certain developmental phases of pregnancy, when it then displays its harmful effects (Mason et al. 2014). Additionally, abnormal high Pb accumulation can impair the expression and function of placental transporters, such as amino acid transporters and glucose transporters, which are essential for fetal nutrition and development (Collin et al. 2022).

As a response to the oxidative stress, it may be suggested that the abortion in goats could be attributed to the reduction of essential elements such as P, Fe, and Zn (Casas and Sordo 2006); the reduction of total proteins (Collin et al. 2022) required for the synthesis of progesterone (P_4_); and the alteration in gene expression related to enzymatic and hormonal codes because of the overproduction of ROS (Hernández-Coro et al. 2021).

According to the current findings, P_4_ levels in the blood of aborted goats were significantly reduced and had an inverse relationship with serum levels of Pb. This may be attributed to the partial or complete block of protein synthesis as shown in current data, which consequently reduces the process of protein synthesis needed for progesterone (P_4_) synthesis. This assumption is confirmed by the severe drop in the level of P_4_ in the blood to a level not enough to fix the attachment of embryos to the placenta, causing abortion. This may be attributed to Pb accumulation which might enhance the overproduction of ROS, leading to oxidative damage of the mitochondria and endoplasmic reticulum, which are responsible for energy production and protein synthesis, respectively. In our current data on aborted goats, the threshold level of P_4_ to induce abortion was ≤ 0.48 ng/dL. The inhibition of P_4_ may also be referred to as the toxicity of Pb that alter and/or disturb the gene expression of endogenous antioxidants (Mao et al. 2018). Hamed et al. (2012) reported that Pb caused severe damage to DNA in the brain, liver, kidney, and reproductive tissues, leading to the production of abnormal strands of mRNA that control the synthesis of P_4_, consequently reducing its production. Pb bioaccumulation caused a significant disturbance in DNA molecular structure that, of course, altered the gene expression of mRNA, causing a significant depletion in the synthesis of proteins in the cells and leading to the shortage of protein precursors required for P_4_ synthesis.

In conclusion, the current results affirmed the following:

1. The lead ions accumulated in the serum of aborted goats were significantly higher than those of non-aborted goats.
2. The levels of PAG1 and P_4_ in the blood of goats that gave birth to twins were significantly higher than those of goats that gave birth to single kids, and both were markedly higher than those of aborted goats.
3. In aborted goats, the time of gestation exhibited a significant direct exponential relationship with serum Pb content, accompanied by a significant positive correlation coefficient of + 0.98. In contrast, the levels of serum PAG1 and P_4_ showed a significant inverse power and exponential relationship with the time of gestation, with significant negative correlation coefficients of − 0.78 and − 0.94.
4. The serum Pb content in aborted goats exhibited a significant inverse relationship with each of the PAG1 and P_4_ levels, and these were associated with significant correlation coefficients of − 0.88 and − 0.77 respectively. This indicates that Pb accumulation is the main dependent factor that severely reduces the levels of serum PAGs and P_4_, which in turn causes abortion.
5. The threshold level of serum Pb content required to cause abortion was ≥ 32.08 μg/dl, whereas serum PAG1 and P_4_ were ≤ 0.95 ng/ml and ≤ 0.48 ng/ml, respectively. The threshold levels ≥ 12.34 ng/ml and ≥ 31.52 ng/ml for P_4_ and PAG1, respectively, were needed to deliver twins.
6. PAG1 and P_4_ levels are also key factors in determining whether Zaraibi goats will give birth to twins.
7. The results of the current study shed light on pollutants and the extent of their impact on livestock in the Arab Republic of Egypt, which requires us to carry out more research that must work to combat pollution in all its forms, biologically and chemically, in order to advance livestock, through which we can fill the food gap as well as limit the economic deterioration of livestock, which is considered the food artery for the masses of Egyptian people who suffer from the deterioration of livestock.

Based on our current field studies, we hope that the gentlemen responsible for managing animal farms will follow up on the different kinds of environmental pollution that have horrific effects on the productivity of those farms and work to treat and avoid destructive factors to avoid heavy losses to Egyptian income.

## Data Availability

All data analyzed during the current study are available from the corresponding author on request.

## References

[CR1] Puertollano A, Puertollano ME, Alvarez de Cienfuegos G, de Pablo AM (2011). Dietary Antioxidants: Immunity and Host Defense. Current Topics in Medicinal Chemistry.

[CR2] Aboul-Naga AM, Hamed A, Shaat I, Mabrouk MMS (2012). Genetic improvement of Egyptian Nubian goats as sub-tropical dairy prolific breed. Small Ruminant Research.

[CR3] Agbugui M, Abe GO (2022). Heavy Metals in Fish. Bioaccumulation and Health British Journal of Earth Sciences Research.

[CR4] Alper MM, Seibel MM, Oskowitz SP, Taymor ML (1987). Comparison of follicular fluid hormones in patients with one or two ovaries participating in a program of in vitro fertilization. Fertility and Sterility.

[CR5] Arashiro EKN, Ungerfeld R, Clariget RP, Pinto PHN, Balaro MFA, Bragança GM, Ribeiro LS, da Fonseca JF, Brandão FZ (2018). Early pregnancy diagnosis in ewes by subjective assessment of luteal vascularisation using colour Doppler ultrasonography. Theriogenology.

[CR6] Arck P, Hansen PJ, Jericevic BM, Piccinni MP, Szekeres-Bartho J (2007). Progesterone during pregnancy: Endocrine-immune cross talk in Mammalian Species and the role of stress. American Journal of Reproductive Immunology.

[CR7] Austin KJ, King CP, Vierk JE, Sasser RG, Hansen TR (1999). Pregnancy-specific protein B induces release of an alpha chemokine in bovine endometrium. Endocrinology.

[CR8] Azeh Engwa, G., Udoka Ferdinand, P., Nweke Nwalo, F. and N. Unachukwu, M., 2019. Mechanism and Health Effects of Heavy Metal Toxicity in Humans. 10.5772/INTECHOPEN.82511

[CR9] Bak A, Roszkowski K (2013). Oxidative stress in pregnant women. Archives of Perinatal Medicine.

[CR10] Barbato O, Menchetti L, Brecchia G, Barile VL (2022). Using Pregnancy-Associated Glycoproteins (PAGs) to Improve Reproductive Management: From Dairy Cows to Other Dairy Livestock Animals.

[CR11] Barry PSI (1975). A comparison of concentrations of lead in human tissues. British Journal of Industrial Medicine.

[CR12] Batalha ES, Sulon J, Figueiredo JR, Beckers JF, Espeschit CJB, Martins R, Silva LDM (2001). Plasma profile of pregnancy associated glycoprotein (PAG) in pregnant alpine goats using two radioimmunoassay (RIA) systems. Small Ruminant Research.

[CR13] Belyaeva EA, Dymkowska D, Wieckowski MR, Wojtczak L (2008). Mitochondria as an important target in heavy metal toxicity in rat hepatoma AS-30D cells. Toxicology and Applied Pharmacology.

[CR14] Bíreš J, Dianovský J, Bartko P, Juhásová Z (1995). Effects on enzymes and the genetic apparatus of sheep after administration of samples from industrial emissions. Biometals.

[CR15] Bouayed J, Bohn T (2010). Exogenous antioxidants - Double-edged swords in cellular redox state: Health beneficial effects at physiologic doses versus deleterious effects at high doses. Oxidative Medicine and Cellular Longevity.

[CR16] Canaz E, Kilinc M, Sayar H, Kiran G, Ozyurek E (2017). Lead, selenium and nickel concentrations in epithelial ovarian cancer, borderline ovarian tumor and healthy ovarian tissues. Journal of Trace Elements in Medicine and Biology.

[CR17] Casas, J.A.S. and Sordo, J.A., 2006. Lead Lead, 158–228. 10.1186/s12889-016-3902-3

[CR18] Chentouf M, El Amiri B, Sulon J, Beckers JF, Kirschvink N, Boulanouar B, Bister JL (2008). Pregnancy-associated glycoprotein secretion in North Moroccan goats. Reproduction in Domestic Animals.

[CR19] Collin, M.S., Venkatraman, S.K., Vijayakumar, N., Kanimozhi, V., Arbaaz, S.M., Stacey, R.G.S., Anusha, J., Choudhary, R., Lvov, V., Tovar, G.I., Senatov, F., Koppala, S. and Swamiappan, S., 2022. Bioaccumulation of lead (Pb) and its effects on human: A review Journal of Hazardous Materials Advances 10.1016/j.hazl.2022.100064

[CR20] Convey EM (1974). Serum Hormone Concentration in Ruminants during Mammary Growth. Lactogenesis, and Lactation: A Review Journal of Dairy Science.

[CR21] Dasharathy, S., Arjunan, S., Maliyur Basavaraju, A., Murugasen, V., Ramachandran, S., Keshav, R. and Murugan, R., 2022. Mutagenic, Carcinogenic, and Teratogenic Effect of Heavy Metals Evidence-based Complementary and Alternative Medicine, 2022. 10.1155/2022/8011953 10.1155/2022/8011953 PMC9556253 36248437

[CR22] Dinny Graham J, Clarke CL (1997). Physiological action of progesterone in target tissues. Endocrine Reviews.

[CR23] Dosogne H, Burvenich C, Freeman AE, Kehrli ME, Detilleux JC, Sulon J, Beckers JF, Hoeben D (1999). Pregnancy-associated glycoprotein and decreased polymorphonuclear leukocyte function in early post-partum dairy cows. Veterinary Immunology and Immunopathology.

[CR24] Elarabany N, El-Batrawy O (2019). Physiological changes in the Cattle Egret, Bubulcus ibis, as a bioindicator of air pollution in New Damietta City, Egypt African. Journal of Biological Sciences.

[CR25] El-Tohamy MM, Hamam AM, Ali UA (1997). Reproductive efficiency of buffalo-cows and its relationship with some heavy metals in the soil. Egypt. J. Applied Sci..

[CR26] Faye D, Sulon J, Kane Y, Beckers JF, Leak S, Kaboret Y, De Sousa NM, Losson B, Geerts S (2004). Effects of an experimental Trypanosoma congolense infection on the reproductive performance of West African Dwarf goats. Theriogenology.

[CR27] Filho RVO, Franco GA, Reese ST, Dantas FG, Fontes PLP, Cooke RF, Rhinehart JD, Thompson KW, Pohler KG (2020). Using pregnancy associated glycoproteins (PAG) for pregnancy detection at day 24 of gestation in beef cattle. Theriogenology.

[CR28] Gaafar KM, Gabr MK, Teleb DF (2005). The hormonal profile during the estrous cycle and gestation in Damascus goats. Small Ruminant Research.

[CR29] Galal S (2005). Biodiversity in goats. Small Ruminant Research - SMALL RUMINANT RES..

[CR30] Garbayo JM, Serrano B, Lopez-Gatius F (2008). Identification of novel pregnancy-associated glycoproteins (PAG) expressed by the peri-implantation conceptus of domestic ruminants. Animal Reproduction Science.

[CR31] Gensa, U., 2019. Review on Cyanide Poisoning in Ruminants. Journal of Biology, Agriculture and Healthcare, 9(6), 1–12. 10.7176/JBAH

[CR32] Ghazzal, M., Hussain, M.I., Khan, Z.I., Habib ur Rahman, M., El-Habeeb, A.A. and Yang, H.H., 2022. Chromium Poisoning in Buffaloes in the Vicinity of Contaminated Pastureland, Punjab, Pakistan Sustainability (Switzerland), 14; 10.3390/su142215095

[CR33] González F, Cabrera F, Batista M, Rodríguez N, Álamo D, Sulon J, Beckers JF, Gracia A (2004). A comparison of diagnosis of pregnancy in the goat via transrectal ultrasound scanning, progesterone, and pregnancy-associated glycoprotein assays. Theriogenology.

[CR34] González F, Sulon J, Garbayo JM, Batista M, Cabrera F, Calero PO, Gracia A, Beckers JF (2000). Secretory profiles of pregnancy-associated glycoproteins at different stages of pregnancy in the goat. Reproduction in Domestic Animals.

[CR35] Goutam Mukherjee A, Ramesh Wanjari U, Renu K, Vellingiri B, Valsala Gopalakrishnan A (2022). Heavy metal and metalloid - induced reproductive toxicity. Environmental Toxicology and Pharmacology.

[CR36] Graziano JH, Blum CB, Lolacono NJ, Slavkovich V, Manton WI, Pond S, Moore MR (1996). A human in vivo model for the determination of lead bioavailability using stable isotope dilution. Environmental Health Perspectives.

[CR37] Green JA, Xie S, Quan X, Bao B, Gan X, Mathialagan N, Beckers F, Roberts RM (2000). Pregnancy-associated bovine and ovine glycoproteins exhibit spatially and temporally distinct expression patterns during pregnancy. Biology of Reproduction.

[CR38] Gulson BL, Jameson CW, Mahaffey KR, Mizon KJ, Patison N, Law AJ, Korsch MJ, Salter MA (1998). Relationships of lead in breast milk to lead in blood, urine, and diet of the infant and mother. Environmental Health Perspectives.

[CR39] Haile A (2014). Assessment of Major Reproductive Disorders of Dairy Cattle in Urban and Per Urban Area of Hosanna. Southern Ethiopia Animal and Veterinary Sciences.

[CR40] Hajiabadi N, Fathi E, Hamli H (2022). Relationships between hematological parameters and Cl and Na homeostasis in dairy herds and abortion. International journal of health sciences.

[CR41] Hamed, M.A., Ali, S.A. and Saba El-Rigal, N., 2012. Therapeutic potential of ginger against renal injury induced by carbon tetrachloride in rats The Scientific World Journal. 10.1100/2012/840421 10.1100/2012/840421 PMC3329925 22566780

[CR42] Hassen AS, Tesfaye Y (2014). Sheep and goat production objectives in pastoral and agro-pastoral production systems in Chifra district of Afar, Ethiopia. Tropical Animal Health and Production.

[CR43] Hernández-Coro A, Sánchez-Hernández BE, Montes S, Martínez-Lazcano JC, González-Guevara E, Pérez-Severiano F (2021). Alterations in gene expression due to chronic lead exposure induce behavioral changes. Neuroscience and Biobehavioral Reviews.

[CR44] Hosny, H., Aziz, A., Shawky, H. and Harby, A.A., 2022. The economics of sheep and goat meat production in Egypt: a case study in Gharbia Governorate. IOSR Journal of Economics and Finance (IOSR-JEF), 13(2), 07–17.

[CR45] Hu H, Rabinowitz M, Smith D (1998). Bone lead as a biological marker in epidemiologic studies of chronic toxicity: Conceptual paradigms. Environmental Health Perspectives.

[CR46] Huang N, Pandey AV, Agrawal V, Reardon W, Lapunzina PD, Mowat D, Jabs EW, Van Vliet G, Sack J, Flück CE, Miller WL (2005). Diversity and function of mutations in P450 oxidoreductase in patients with Antley-Bixler syndrome and disordered steroidogenesis. American Journal of Human Genetics.

[CR47] Karadaev M (2015). Pregnancy diagnosis techniques in goats – A review. Bulgarian Journal of Veterinary Medicine.

[CR48] Kolatorova, L., Vitku, J., Suchopar, J., Hill, M. and Parizek, A., 2022. Progesterone: A Steroid with Wide Range of Effects in Physiology as Well as Human Medicine International Journal of Molecular Sciences, 23. 10.3390/ijms23147989 10.3390/ijms23147989 PMC9322133 35887338

[CR49] Lonergan P (2011). Influence of progesterone on oocyte quality and embryo development in cows. Theriogenology.

[CR50] Mao T, Han C, Wei B, Zhao L, Zhang Q, Deng R, Liu J, Luo Y, Zhang Y (2018). Protective Effects of Quercetin Against Cadmium Chloride-Induced Oxidative Injury in Goat Sperm and Zygotes. Biological Trace Element Research.

[CR51] Mason, L.H., Harp, J.P. and Han, D.Y., 2014. Pb neurotoxicity: Neuropsychological effects of lead toxicity BioMed Research International, 2014. 10.1155/2014/840547 10.1155/2014/840547 PMC3909981 24516855

[CR52] Massányi P, Massányi M, Madeddu R, Stawarz R, Lukáč N (2020). Effects of cadmium, lead, and mercury on the structure and function of reproductive organs. Toxics.

[CR53] McEvoy JD, McCoy M (1993). Acute lead poisoning in a beef herd associated with contaminated silage. Veterinary Res..

[CR54] Moore TA, Ahmad IM, Schmid KK, Berger AM, Ruiz RJ, Pickler RH, Zimmerman MC (2019). Oxidative Stress Levels Throughout Pregnancy, at Birth, and in the Neonate Biological Research for. Nursing.

[CR55] Morecroft CW, Mackridge AJ, Stokes EC, Gray NJ, Wilson SE, Ashcroft DM, Mensah N, Pickup GB (2015). Involving community pharmacists in pharmacy practice research: experiences of peer interviewing. International Journal of Clinical Pharmacy.

[CR56] Morrow PE, Beiter H, Amato F, Gibb FR (1980). Pulmonary retention of lead: An experimental study in man. Environmental Research.

[CR57] Morsy GM, El-ala KSA, Ali AA (2016). Studies on fate and toxicity of nanoalumina in male albino rats : Lethality , Studies on fate and toxicity of nanoalumina in male albino rats : Lethality , bioaccumulation and genotoxicity. Toxicology and industrial health.

[CR58] Mridula, Guvvala N, Sarathi R, Vinu R (2022). Effect of Zeolite Addition on Partial Discharge and Dielectric Behavior of Thermally Aged Synthetic Ester Fluid Under External Magnetic Field. IEEE Access.

[CR59] Nowier AM, Darwish HR, Ramadan SI, Othman OE (2020). Polymorphism of lactoferrin gene in Egyptian goats and its association with milk composition traits in Zaraibi breed. Tropical Animal Health and Production.

[CR60] Padilla-Rivas GR, Sohnrey B, Holtz W (2005). Early pregnancy detection by real-time ultrasonography in Boer goats. Small Ruminant Research.

[CR61] Panasiewicz G, Lipka A, Majewska M, Bieniek-Kobuszewska M, Saveljev AP, Szafranska B (2019). Identification of pregnancy-associated glycoprotein family (PAG) in the brown bear (Ursus arctos L.). Acta Histochemica.

[CR62] Pohler KG, Franco GA, Reese ST, Dantas FG, Ellis MD, Payton RR (2016). Past, present and future of pregnancy detection methods. Applied Reproductive Strategies in Beef Cattle.

[CR63] Quintela LA, Barrio M, Peña AI, Becerra JJ, Cainzos J, Herradón PG, Díaz C (2012). Use of Ultrasound in the Reproductive Management of Dairy Cattle. Reproduction in Domestic Animals.

[CR64] Rădulescu A, Lundgren S (2019). A pharmacokinetic model of lead absorption and calcium competitive dynamics. Scientific Reports.

[CR65] Rawlings NC, Ward WR (1977). Progesterone and the initiation of parturition in the goat. Theriogenology.

[CR66] Roberts JN, May KJ, Veiga-Lopez A (2017). Time-dependent changes in pregnancy-associated glycoproteins and progesterone in commercial crossbred sheep. Theriogenology.

[CR67] Salve RR, Ingole SD, Nagvekar AS, Bharucha SV, Dagli NR (2016). Pregnancy associated protein and progesterone concentrations during early pregnancy in Sirohi goats. Small Ruminant Research.

[CR68] Sawada T, Nakatani T, Tamada H, Mori J (1994). Secretion of progesterone and 20 alpha-dihydroprogesterone during pregnancy in goats. Steroids.

[CR69] Smith CJ, Denes A, Tyrrell PJ, Di Napoli M (2015). Phase II anti-inflammatory and immune-modulating drugs for acute ischaemic stroke. Expert Opinion on Investigational Drugs.

[CR70] Sousa NM, Garbayo JM, Figueiredo JR, Sulon J, Gonçalves PBD, Beckers JF (1999). Pregnancy-associated glycoprotein and progesterone profiles during pregnancy and postpartum in native goats from the north-east of Brazil. Small Ruminant Research.

[CR71] Stauber JL, Florence TM, Gulson BL, Dale LS (1994). Percutaneous absorption of inorganic lead compounds. Science of the Total Environment.

[CR72] Thorburn GD, Schneider W (1972). The progesterone concentration in the plasma of the goat during the oestrous cycle and pregnancy. The Journal of endocrinology.

[CR73] Toboła-Wróbel, K., Pietryga, M., Dydowicz, P., Napierała, M., Brązert, J. and Florek, E., 2020. Association of Oxidative Stress on Pregnancy Oxidative Medicine and Cellular Longevity, 2020. 10.1155/2020/6398520 10.1155/2020/6398520 PMC7512072 33014274

[CR74] Stoev SD, Manov V, Vassilev N (1997). Morphological investigation in experimental cases of chronic lead poisoning in pregnant sheep Bulgarian. J. Agric. Sci..

[CR75] Vasques MI, Horta AEM, Marques CC, Sasser RG, Humblot P (1995). Levels of bPSPB throughout single and twin pregnancies after AI or transfer of IVM/IVF cattle embryos. Animal Reproduction Science.

[CR76] Volkov R, Ezhkova A (2020). Migration of heavy metals in the system “soil-plant-animal-livestock products”. BIO Web of Conferences.

[CR77] Woo DK, Shadel GS (2011). Mitochondrial stress signals revise an old aging theory. Cell.

[CR78] World Health Organization, 1987. WHO advisory committee on variola virus research: report of the seventeenth meeting, Geneva, Switzerland, 12–13.

[CR79] Wright, R.O., Tsaih, S.W., Schwartz, J., Wright, R.J. and Hu, H., 2003. Association between iron deficiency and blood lead level in a longitudinal analysis of children followed in an urban primary care clinic Journal of Pediatrics, 142, 9–14. 10.1067/mpd.2003.mpd0344 10.1067/mpd.2003.mpd0344 12520247

[CR80] Xie S, Low BG, Nagel RJ, Beckers JF, Roberts RM (1994). A novel glycoprotein of the aspartic proteinase gene family expressed in bovine placental trophectoderm. Biology of Reproduction.

[CR81] Xie S, Low BG, Nagel RJ, Kramer KK, Anthony RV, Zoli AP, Beckers JF, Roberts RM (1991). Identification of the major pregnancy-specific antigens of cattle and sheep as inactive members of the aspartic proteinase family. Proceedings of the National Academy of Sciences of the United States of America.

[CR82] Yadav, R., Yadav, P., Singh, G., Kumar, S., Dutt, R. and Pandey, A., 2021. Non Infectious Causes of Abortion in Livestock Animals -A Review International Journal of Livestock Research, 1. 10.5455/ijlr.20201031

[CR83] Zamfirescu S, Anghel A, Nadolu D, Dobrin N (2011). Plasmatic profiles of pregnancy-associated glycoprotein and progesterone levels during early pregnancy in carpathian goat. Annals of the Romanian Society for Cell Biology.

[CR84] Zarrouk, A., Engeland, I. V., Sulon, J. and Beckers, J.F., 1999. Pregnancy-associated glycoprotein levels in pregnant goats inoculated with Toxoplasma gondii OR Listeria monocytogenes: A retrospective study Theriogenology, 52, 1095–1104. 10.1016/s0093-691x(99)00197-1 10.1016/s0093-691x(99)00197-1 10735115

[CR85] Zoli AP, Guilbault LA, Delahaut P, Ortiz WB, Beckers JF (1992). Radioimmunoassay of a bovine pregnancy-associated glycoprotein in serum: Its application for pregnancy diagnosis. Biology of Reproduction.

